# MiR-23a-5p alleviates chronic obstructive pulmonary disease through targeted regulation of RAGE-ROS pathway

**DOI:** 10.1186/s12931-024-02736-y

**Published:** 2024-02-20

**Authors:** Chenli Chang, Ke Huang, Xia Xu, Ruirui Duan, Tao Yu, Xu Chu, Chen Chen, Baicun Li, Ting Yang

**Affiliations:** 1 China-Japan Friendship Hospital (Institute of Clinical Medical Sciences), Chinese Academy of Medical Sciences & Peking Union Medical College, Beijing, China; 2 Center of Respiratory Medicine, China-Japan Friendship Hospital, National Center for Respiratory Medicine, Institute of Respiratory Medicine, Chinese Academy of Medical Sciences, National Clinical Research Center for Respiratory Diseases, State Key Laboratory of Respiratory Health and Multi Morbidity, No 2, East Yinghua Road, Chaoyang District, Beijing, 100029 China; 3 Department of Immunology, School of Basic Medical Sciences, Capital Medical University, Beijing, China

**Keywords:** COPD, RAGE, miR-23a-5p, ROS, Cigarette smoke

## Abstract

**Background:**

Chronic obstructive pulmonary disease (COPD) is a common respiratory disease and represents the third leading cause of death worldwide. This study aimed to investigate miRNA regulation of Receptor for Advanced Glycation End-products (RAGE), a causal receptor in the pathogenesis of cigarette smoke (CS)-related COPD, to guide development of therapeutic strategies.

**Methods:**

RAGE expression was quantified in lung tissue of COPD patients and healthy controls, and in mice with CS-induced COPD. RNA-sequencing of peripheral blood from COPD patients with binding site prediction was used to screen differentially expressed miRNAs that may interact with RAGE. Investigation of miR-23a-5p as a potential regulator of COPD progression was conducted with miR-23a-5p agomir in COPD mice in vivo using histology and SCIREQ functional assays, while miR-23a-5p mimics or RAGE inhibitor were applied in 16-HBE human bronchial epithelial cells in vitro. RNA-sequencing, ELISA, and standard molecular techniques were used to characterize downstream signaling pathways in COPD mice and 16-HBE cells treated with cigarette smoke extract (CSE).

**Results:**

RAGE expression is significantly increased in lung tissue of COPD patients, COPD model mice, and CSE-treated 16-HBE cells, while inhibiting RAGE expression significantly reduces COPD severity in mice. RNA-seq analysis of peripheral blood from COPD patients identified miR-23a-5p as the most significant candidate miRNA interaction partner of RAGE, and miR-23a-5p is significantly downregulated in mice and cells treated with CS or CSE, respectively. Injection of miR-23a-5p agomir leads to significantly reduced airway inflammation and alleviation of symptoms in COPD mice, while overexpressing miR-23a-5p leads to improved lung function. RNA-seq with validation confirmed that reactive oxygen species (ROS) signaling is increased under CSE-induced aberrant upregulation of RAGE, and suppressed in CSE-stimulated cells treated with miR-23a-5p mimics or overexpression. ERK phosphorylation and subsequent cytokine production was also increased under RAGE activation, but inhibited by increasing miR-23a-5p levels, implying that the miR-23a-5p/RAGE/ROS axis mediates COPD pathogenesis via ERK activation.

**Conclusions:**

This study identifies a miR-23a-5p/RAGE/ROS signaling axis required for pathogenesis of COPD. MiR-23a-5p functions as a negative regulator of RAGE and downstream activation of ROS signaling, and can inhibit COPD progression in vitro and in vivo, suggesting therapeutic targets to improve COPD treatment.

**Graphical Abstract:**

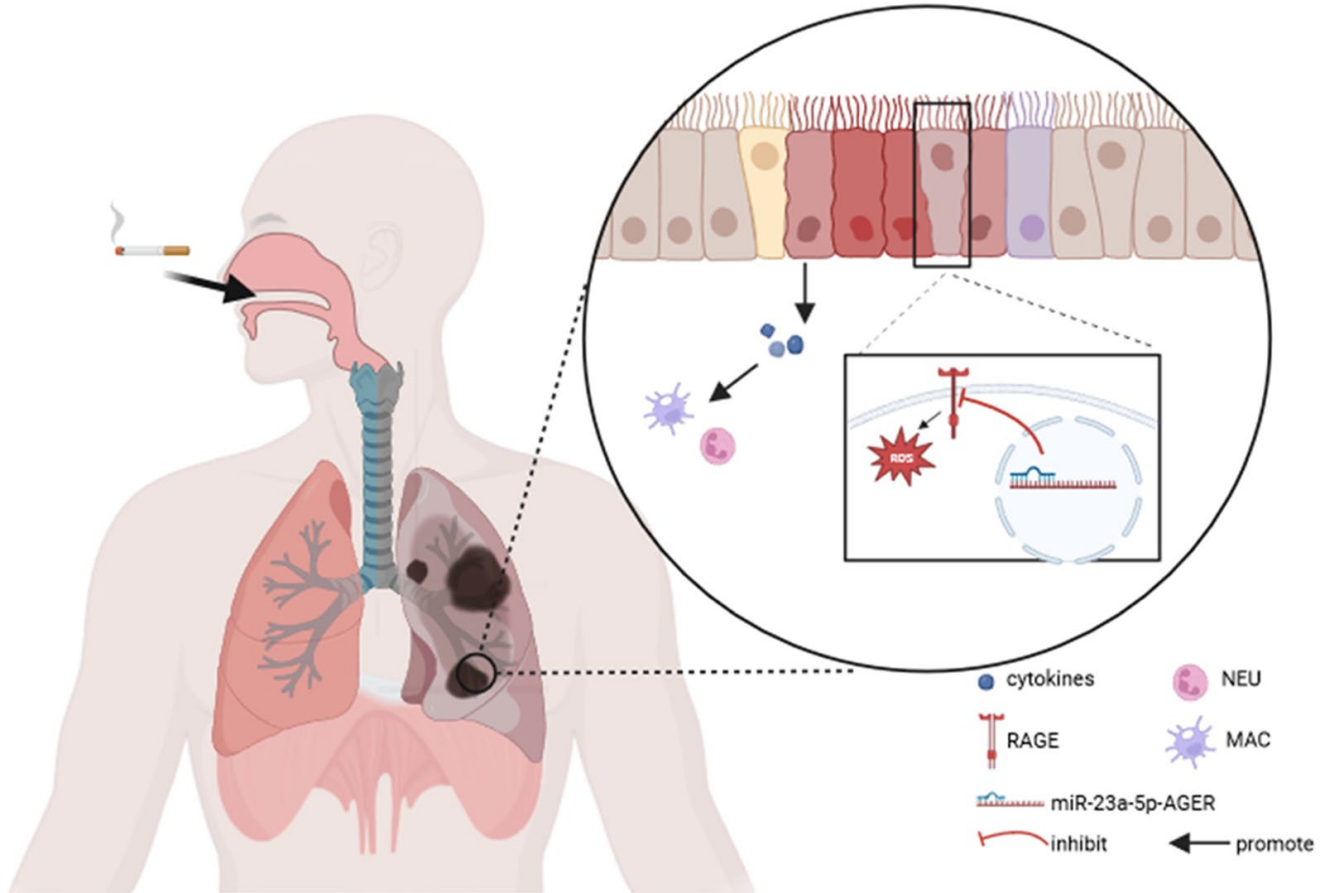

**Supplementary Information:**

The online version contains supplementary material available at 10.1186/s12931-024-02736-y.

## Background

Chronic obstructive pulmonary disease (COPD) is the most common chronic disease of the respiratory system, characterized by persistent respiratory symptoms and limitation of airflow. Its main pathological features include chronic airway inflammation, airway remodeling, and emphysema, and thus posing a serious danger to public health [1, 2], with the latest study of Global Burden of Disease (GBD) identifying COPD as the third leading cause of death worldwide [3]. In previous work, our team spearheaded the China Adult Pulmonary Health Study (CPHS) survey, revealing a COPD prevalence of 13.7% among people aged 40 and above in China. Based on national census data, an estimated 100 million individuals suffer from COPD in China alone [4], but despite its high socioeconomic burden and mortality rate, our understanding of the mechanisms underlying the onset and progression of this chronic lung ailment remains incomplete. Furthermore, this long-standing gap in knowledge gap poses challenges for identifying new and potentially effective therapeutic targets for this pressing public health issue.

Long-term exposure to cigarette smoke (CS) or second-hand smoke is among the main causes of COPD, and they have been firmly linked through experimental evidence [5]. Cigarette smoke can induce inflammatory responses in various tissues, and especially in the airway epithelium. This inflammatory response can further lead to sustained recruitment of immune cells, squamous metaplasia, high mucus secretion, cilia shedding, and limited airflow [6]. Additionally, oxidative stress induced by cigarette smoke can disrupt connections between adjacent epithelial cells, which plays a crucial role in the development of emphysema [7–9].

Among the molecular genetic factors contributing to COPD, previous studies have identified Receptor for Advanced Glycation End-products (RAGE) as a central component in the development of inflammation in COPD [5, 10]. As a transmembrane pattern recognition receptor, RAGE can bind to numerous endogenous ligands, such as HMGB1, S100, HSP70, etc., respectively activating MAPK/NF-κB, Ras, PI3K-AKT, and other signaling pathways that stimulate the release of inflammatory mediators, cytokines, and chemokines, which in turn trigger a strong inflammatory cascade that ultimately leads to tissue destruction and organ damage [11–14]. Previous genome-wide association studies (GWAS) have identified RAGE as a susceptibility gene for COPD [15]. Furthermore, RAGE overexpression in mouse bronchial-alveolar lavage fluid (BALF)  upregulates the expression of inflammatory factors, accelerates epithelial cell apoptosis, and consequently increasing susceptibility to emphysema [16]. Notably, the extent of emphysema formation, abundance of infiltrating inflammatory immune cells, and inflammatory factor levels in BALF were all significantly lower in RAGE knockout mice compared to wild-type controls exposed to cigarette smoke [17]. Specific antagonist of RAGE can inhibit inflammatory cell aggregation, inflammatory factor secretion, and expansion of alveolar spaces induced by elastase [18]. Alternatively, cigarette smoke can induce RAGE expression in alveolar epithelial cells, which subsequently contributes to lung inflammation [19]. Previous work by our research group revealed that RAGE mRNA levels in peripheral blood of COPD patients are negatively correlated with FEV_1_, an indicator of lung function [20]. However, the mechanism by which increased RAGE expression leads to COPD pathogenesis has not been fully determined, while the upstream regulatory mechanism responsible for its activation remains unclear.

MiRNAs are non-coding, single-stranded, 20–24 nucleotide (nt) small RNAs that are known to inhibit translation or mediate degradation of target gene mRNAs through binding to a 6–8 nt or longer complementary sequence in the 3′ untranslated region (3'UTR). This post-transcriptional regulation has been identified in cell differentiation, apoptosis, and proliferation among other fundamental cellular processes [21]. Studies examining miRNA expression in lung tissue, specific cell types in the lung, peripheral blood, and immune cells have identified many miRNAs associated with COPD and/or COPD-related phenotypes, such as emphysema and asthma-COPD overlap syndrome [22–25]. The effects of smoking and cigarette smoke components on miRNA expression and function are well-established as central features of COPD research, especially studies aiming to elucidate the relationship between exposure to cigarette smoke and non-coding RNAs (including miRNAs), their role in COPD pathogenesis, and new therapeutic targets for treating COPD.

Due to the lack of studies on miRNA regulation of RAGE in COPD, we screened bulk RNAseq data from COPD patients and healthy volunteers and found that miR-23a-5p is down-regulated, while RAGE is aberrantly upregulated, in COPD, which led to our hypothesis that RAGE may be a regulatory target of miR-23a-5p. Using a cigarette smoke (CS)-induced murine model of COPD and human bronchial epithelial (16-HBE) cells, we explored the relationship between miR-23a-5p and RAGE and its role in the pathogenesis of COPD. Our results confirmed that miR-23a-5p is downregulated while RAGE is upregulated following CS exposure. Increased RAGE expression activates downstream ROS-mediated signaling, which in turn promotes infiltration of inflammatory cells and production of inflammatory cytokines. Alternatively, overexpressing miR-23a-5p or applying a miRNA mimic in vitro, or injection with miR-23a-5p agomir in vivo can suppress RAGE upregulation, ROS signaling and downstream inflammatory factor secretion, ultimately conferring therapeutic effects ameliorating damage from lung inflammation and alveolar structural destruction, and inhibiting COPD progression. Our study provides a basis for targeting miR-23a-5p to inhibit RAGE-mediated inflammatory response and alveolar destruction processes in COPD, warranting further exploration as potential therapeutic strategy for COPD.

## Methods

### Subjects

According to a preliminary clinical cohort study, we selected 13 patients with COPD alongside 19 healthy controls. All subjects were from the China-Japan Friendship Hospital, and 13 patients with COPD met the diagnostic criteria: after inhaling bronchodilators, FEV_1_/FVC < 70%. All subjects provided written informed consent. The study received approval from the Ethics Committee of the China-Japan Friendship Hospital (2022-KY-141).

### Animals

Six to 8-week-old male C57BL/6 mice were acquired from Si Pei Fu (Beijing) Biotechnology Co., Ltd. All mice were housed in a controlled, pathogen-free environment in the Animal Experiment Center of the Clinical Medical Research Institute of the China-Japan Friendship Hospital. Mice were housed at room temperature (24 ± 2 °C) under a 12-h light/ 12-h dark cycle and consistent humidity (50 ± 5%). All experimental protocols received approval from the Animal Research Ethics Committee of the China-Japan Friendship Hospital.

### In vivo CS exposure

The CS-exposed mice were placed into the exposure chamber, and an automatic lighter was used to light the cigarette. A vacuum extractor was employed to inhale the smoke into the exposure chamber. Each exposure consisted of 20 cigarettes, and the particle concentration in the chamber was maintained at 80–120 mg/m^3^. The exposure lasted for 60 min, 4 times daily, five days per week. The control group was exposed to fresh air during each session, and this experiment lasted 24 weeks. All mice were fed a normal diet with a 12-h light/ 12-h dark cycle.

### Mouse lung function examination

Weights were examined in mice after exposure to sufficient modeling time, and a corresponding volume of 2% pentobarbital sodium intraperitoneal injection (5 µL/g) was administered. Following anesthesia, the mice were subjected to tracheal intubation. The SCIREQ small animal invasive lung function instrument was used to examine the lung function of the mice, including measurements of deep inspiratory volume (IC), main airway resistance (Rn), and 0.1-s rate (FEV_0.1_/FVC).

### Collection of alveolar lavage fluid

Following lung function testing in mice, 0.8 mL of PBS was injected into both lungs via an air tube and rinsed 5 times. The aspirated lavage solution was centrifuged at 4 ℃ for 10 min, and the resulting supernatant was stored at − 80 ℃ for cytokine assessment. A sample of 1 mL PBS containing 1% BSA was used to resuspend the cell precipitate, and 10 μL of the cell suspension was obtained for cell counting. The cell concentration was adjusted after cell counting. A Cytospin centrifuge (Thermofisher, USA) was used to obtain the cells, which were fixed with 4% paraformaldehyde following air drying (Servicebio, Wuhan). Cell slides were stained using hematoxylin and eosin (H&E) for classification and counting.

### Pathological staining of mouse lung tissue

Mouse lung tissue was fixed using 4% paraformaldehyde for 24 h. After fixation, the lungs were embedded in paraffin for H&E examination. The mean lining interval (MLI) was determined based on previous studies.

### Luciferase activity assessment

Control plasmids (Genepharm, China) and plasmids carrying the AGER 3′-UTR of wild-type and mutant were co-transfected in conjunction with the miR-23a-5p mimic and a non-targeting control miRNA, respectively, into 293T cells. Subsequent to a 24-h incubation, harvest, and lysis, and the cells’ chemiluminescence intensity was examined by using a Dual-Luciferase Reporter Assay Kit (Promega, USA). The activity of firefly luciferase was standardized with *Renilla* luciferase as reference.

### Fluorescence in situ hybridization (FISH)

Based on the manufacturer's instructions from RNAWeAMI ™, an in situ hybridization multiple fluorescence detection kit (Xavier Biotechnology Co., Ltd.) was used for RNA FISH. An assembly of dual-tagged probes targeting RAGE and miR-23a-5p was designed, and FISH was conducted on paraffinized sections of mouse lung tissue. Deparaffinization was performed using a series consisting of continuous xylene, graded ethanol, and deionized water, followed by antigen thermal repair and digestion using proteinase K and pretreatment with hydrogen peroxide solutions. Following pre-hybridization treatment, multiple target probe 1 hybridization, multiple target probe 2 hybridization, and multiple fluorescence probe hybridization (RAGE/IF488, miR-23a-5p/IF550) were conducted sequentially. After DAPI staining of the nuclei, cells were observed using a fluorescence microscope (Nikon). Green fluorescence indicated positive staining with RAGE, while red fluorescence indicated positive staining with miR-23a-5p. The mean fluorescence intensity (MFI) analysis was conducted using ImageJ software (NIH).

### Immunofluorescence

16-HBE cells were inoculated onto the cell slides (NEST, Wuxi) within a 24-well plate and stimulated using CSE for 24 h. The slides were immersed in PBS three times in the culture plate, for 3 min each time. The slide was then fixed with 4% paraformaldehyde for 15 min and soaked in PBS. Cell slides were washed in 0.5% Triton X-100 (PBS preparation) at room temperature for 20 min before being transferred to PBS. Water-absorbent paper was used to dry the PBS, and 10% donkey serum was added at room temperature for 30 min. Following this, a sufficient amount of 1:100 anti-RAGE antibody (Abcam, ab216329) was added onto each slide and placed in an incubation chamber. After incubation at 4 °C overnight, the slides were immersed in PBST, and diluted 1:200 anti-rabbit secondary antibody (abbkine) was added dropwise. The slides were incubated at room temperature for 1 h. An anti-fluorescence quenching sealing agent containing DAPI was used for sealing, and immunohistochemical images were captured using a Nikon microscope (ECLIPSE Nikon). Image analysis was conducted using ImageJ software (NIH). RAGE expression levels were compared based on the average fluorescence intensity.

### Immunohistochemistry

After paraffinized mouse lung tissue sections were dewaxed, antigen repair was conducted using Tris–EDTA buffer (pH 9.0). After treatment with a 3% hydrogen peroxide solution, the sections were sealed at room temperature using an immunostaining blocking solution (Biyuntian) for 30 min. A dilution of 1:1000 anti-RAGE antibodies (Abcam, ab216329) was dripped onto paraffin sections and incubated overnight at 4 °C. Following immersion in HRP-labeled anti-rabbit secondary antibody (Beijing Guanxingyu Technology Co., Ltd.) was added, and the slices were incubated at room temperature for 1 h. The color rendering was performed using the DAB method, and the cells were stained using hematoxylin. The slices were dehydrated and preserved using neutral balsam. Immunohistochemical images were acquired using a fully automatic scanning machine (3D HISTECH, Hungary) and analyzed using ImageJ software (NIH). The expression levels of RAGE were analyzed by ImageJ (NIH) for proportion of positive signal area.

### rt-qPCR

After employing the TriZol-chloroform-isopropanol method to extract RNA from cells and lung tissues, reverse transcription was conducted using a Takara cDNA one-step synthesis kit (Takara, RR036A). Subsequently, qPCR was conducted using a StepOne Plus real-time PCR system (Takara, RR420A). All primers were obtained from Shanghai Sheng Gong Biotechnology Co., Ltd., China. All mRNA expression values were expressed using GUS or β-Actin as internal references for comparison, and the 2 ^− ΔΔ CT^ method was used for analysis. The primers used in qPCR are outlined in Table 1.

**Table 1:** The sequences of primers in this study

Primer	Sequence
H GUSB F	TACGAACGGGAGGTGATCCT
H GUSB R	CCCTCATGCTCTAGCGTGTC
H IL-6 F	CCACCGGGAACGAAAGAGAA
H IL-6 R	TCTCCTGGGGGTATTGTGGA
H IL-8 F	AGTTTTTGAAGAGGGCTGAGA
H IL-8 R	ACCAAGGCACAGTGGAACAA
H IL-1B F	CCAAACCTCTTCGAGGCACA
H IL-1B R	AGCCATCATTTCACTGGCGA
H TNFa F	CTGGGCAGGTCTACTTTGGG
H TNFa R	CTGGAGGCCCCAGTTTGAAT
U6 RT	GTCGTATCCAGTGCAGGGTCCGAGGTATTCGCACTGGATACGACAAAATA
U6 F	AGAGAAGATTAGCATGGCCCCTG
m RAGE F	ACAGAAACCGGCGATGAGG
m RAGE R	TCTCCGCTTCCTCTGACTGA
m GAPDH F	CCCTTAAGAGGGATGCTGCC
m GAPDH R	ACTGTGCCGTTGAATTTGCC
m ACTB F	GCAGGAGTACGATGAGTCCG
m ACTB R	ACGCAGCTCAGTAACAGTCC
H miR-23a-5p F	GGGGGTTCCTGGGGATG
H miR-23a-5p R	GTCGTATCCAGTGCAGGGTCCGAGGTATTCGCACTGGATACGACAAATCC
M IL-6 F	CCCCAATTTCCAATGCTCTCC
M IL-6 R	CGCACTAGGTTTGCCGAGTA
M IL-8 F	CGCCACGTTCTGACCACTTA
M IL-8 R	GAGAGGCATCCGGTTCACAG
M TNF-a F	ACCCTCACACTCACAAACCA
M TNF-a R	ACAAGGTACAACCCATCGGC
M IL-1B F	GAAATGCCACCTTTTGACAGTG
M IL-1B R	TGGATGCTCTCATCAGGACAG

### Western blotting

The lysates of 16-HBE cells and lung tissues following cigarette smoke exposure were prepared using RIPA buffer (Beyotime, P0013B) containing cOmplete™ Protease Inhibitor Cocktail (Roche Diagnostics GmbH, 4693132001-20) and phosphatase inhibitors (Roche Diagnostics GmbH, 04-906-837-001). The total protein concentration was measured using a BCA protein assay kit (ThermoFisher, USA). Proteins were isolated using 10% SDS-PAGE and transferred onto PVDF membranes. They were incubated overnight with anti-RAGE (sc-74473, Santa Cruz Biotechnology, USA), anti-phospho-ERK (ABP0035, abbkine, Wuhan), anti-ERK (ABP0085, abbkine, Wuhan), anti-β-actin (E-AB-48018, elabscience, Wuhan), and anti-GAPDH (5174S, CST, USA) antibodies at 4 ℃. Following washing, the PVDF membranes were incubated with an enzyme-linked secondary antibody for 1 h at room temperature. Quantitative analysis was performed using density measurement alongside ImageJ software.

### ELISA

Mouse alveolar lavage fluid and lung tissue homogenate were acquired, and the levels of IL-6 (Thermo Fisher, 88-7064-88) and IL-1 β (88-7013A-88) were detected according to the manufacturer’s instructions. An enzyme-linked immunosorbent assay (Tecan) was then used to detect the absorbance at 450 nm and 570 nm. A standard curve was prepared according to the instructions, and the concentration of cytokines in the sample was determined.

### Flow cytometry

The upper lobe of the right lung was harvested from mice and processed into a single-cell suspension through physical shredding and enzymatic digestion. Following counting, the cells were divided into two groups and utilized for staining of macrophages and neutrophils, respectively. Macrophages were labeled with live/read, CD45, CD11B, F4-80, CD80, and CD206, while neutrophils were labeled with live/read, CD45, CD11B, CD11C, Ly6G, and SiglecF. Antibodies were from ThermoFisher.

### In vitro cell culture

Human 16-HBE airway epithelial cells were cultured in vitro using RPMI 1640 (Gibco) medium. The cells were changed the following day and passaged on the third day.

### CSE extraction

CSE was prepared for immediate use, using a vacuum extractor for cigarette smoke extraction. Each cigarette (Marlboro, USA) was extracted using 10 mL of RPMI 1640 culture medium, with a fixed extraction duration of 5 min. The absorbance of the liquid was measured at 320 nm using an enzyme-linked immunosorbent assay (Tecan). When the liquid absorbance of 200 μL was found to be 1.0, it was considered 100% CSE, and subsequent dilutions were performed based on this.

### Measurement of reactive oxygen species assay (ROS)

The extracellular and intracellular levels intracellular levels of ROS were measured by CheKine™ Reactive Oxygen Species (ROS) Detection Fluorometric Assay Kit (Abbkine Scientific, Wuhan). In brief, following treatment with CSE and/or other reagents, 5 × 10^5^/mL cells were incubated with a 10 µM solution of DCFH-DA dissolved in serum-free medium at 37 °C for 30 min in a dark condition. Subsequently, cells were washed three times with serum-free medium, then cells were transferred onto the slide using cytospin and images were captured using a Nikon Eclipse fluorescence microscope (Nikon, Japan).

### Complete RNAseq assessment

#### RNA isolation

To isolate the complete RNA content from tissues, we followed the manufacturer’s guidelines employing Trizol (Invitrogen, USA). Approximately 60 mg of ground tissues were homogenized for 2 min before 5 min horizontal standing. Subsequently, the mixture underwent centrifugation (12,000*g*, 4 ℃, 5 min), and the resulting supernatant was relocated to a fresh tube along with 0.3 mL of chloroform/isoamyl alcohol (24:1). The combination was vigorously agitated for 15 s before centrifugation (12,000*g*, 4 ℃, 10 min). The centrifuged aqueous phase (containing the complete RNA) was transferred to another tube along with an same volume of supernatant and isopropyl alcohol. This new mixture underwent centrifugation (12,000*g*, 20 min, 4 ℃) and supernatant removal, the RNA pellet was obtained. After twice rinse with 75% ethanol, the pellet was dried in a biosafety cabinet (about 5–10 min), and ultimately, DEPC-treated water (25–100 µL) was supplemented for RNA dissolution. The overall RNA content was assessed and quantified through the use of a NanoDrop and Agilent 2100 Bioanalyzer (Thermo Fisher, USA).

#### Construction of mRNA Library

mRNA was purified magnetic beads linked to oligo(dT), fragmented into smaller pieces using fragment buffer at 4 ℃, and then was reverse transcribed to first-strand cDNA with a random hexamer primer. Subsequently, the second-strand cDNA was synthesized. An RNA Index Adapters and A-Tailing Mix were supplemented through an incubation step to facilitate end repair. The cDNA fragments from the preceding step were amplified via PCR, and then the fragments were purified using Ampure XP Beads before being dissolved in EB solution, and qualified on an Bioanalyzer (Agilent Technologies 2100). The double-stranded cDNA was subjected to heating, denaturation, and circularization with the help of a splint oligo sequence to create the final library. To compile the eventual library, single-stranded circular DNA (ssCir DNA) was employed. The ultimate library was amplified using phi29 to produce a DNA nanoball (DNB) containing over 300 copies of a single molecule. These DNBs were loaded onto the patterned nanoarray, and paired-end 100 bp reads were obtained using the DNBseq platform (BGI-Shenzhen, China).

### Statistical analysis

Results were examined using one-way analysis of variance (ANOVA) or non-parametric *t*-tests. The Pearson correlation coefficient was utilized to analyze the correlation between continuous variables. A *p*-value of less than 0.05 was considered to be significant. All experiments were repeated in at least triplicate.

## Results

### Exposure to cigarette smoke (CS) is associated with increased RAGE expression

**Fig. 1:**
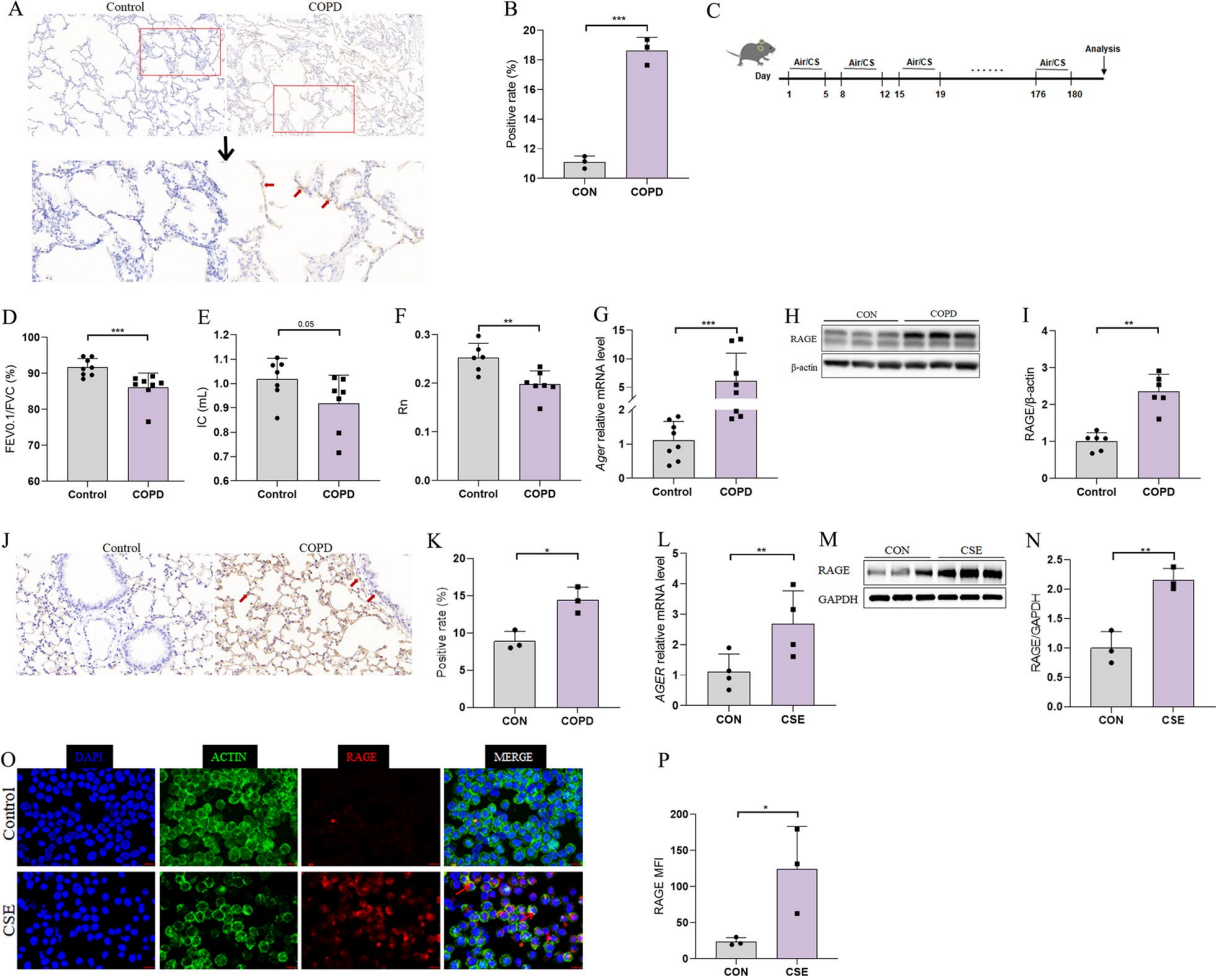
Exposure to cigarette smoke (CS) causes an increase in RAGE expression. **A**, **B** Representative pictures of immunohistochemical staining for RAGE in lung tissues of healthy controls and COPD patients (**A**) and the proportion of positive cells (**B**) (n = 3); **C** strategy for the establishment of the COPD mouse model; **D**–**F** mouse lung function indicators 0.1 s rate (FEV_0.1_/FVC), deep inspiratory volume (IC), and airway Newtonian resistance (Rn) in different groups (n = 6–8); **G** rt-PCR was used to detect the levels of *Ager* mRNA in the lung tissue of control and COPD mice (n = 6–8); **H**, **I** western Blotting was used to detect RAGE protein levels in the lung tissue of control and COPD mice (n = 6–8); **J**, **K** representative immunohistochemical pictures (**J**) and positive rate (**K**) of RAGE in the lung tissue of each group of mice (n = 3); **L** *AGER* mRNA levels in 16-HBE cells were detected by rt-PCR(n = 4). **M**, **N** Western Blotting was used to detect RAGE protein levels in 16-HBE cells (n = 3); **O**, **P** immunofluorescence detection of RAGE positive stains in 16-HBE cell slides (**O**) and MFI analysis (**P**) (n = 3). Data are least squares means ± standard errors. *p < 0.05; **p < 0.01, ***p < 0.001

In light of our previous work that showed RAGE expression was associated with COPD [20], we sought to determine whether and how RAGE played a role in the pathogenesis of COPD. To this end, we collected lung tissue samples from COPD lung transplant patients and non-COPD control subjects for immunohistochemical staining (Fig. 1A, B), which verified that RAGE was significantly elevated in lung tissue of COPD patients (marked by red arrows). We then constructed a mouse model of COPD using the classic 6-month CS exposure method (see scheme in Fig. 1C). H&E staining and measurements of mean liner intercept (MLI) suggested damage to alveolar structure (Additional file 1: Fig. S1A, B). We then used a SCIREQ instrument to examine changes in lung function in CS-exposed mice and found that the FEV_0.1_/FVC ratio (Fig. 1D, p < 0.001) and IC (Fig. 1E, p < 0.01) were significantly lower compared to those parameters in control mice, while the decrease of Rn (Fig. 1F, p = 0.05) was almost statistically significant. Subsequent detection of RAGE mRNA and protein levels were significantly up-regulated in lungs of model mice, similar to our observations in lung samples of COPD patients (Fig. 1G–K).

To further investigate the effect of CS on RAGE expression, we next exposed 16-HBE human bronchial epithelial cells to 2% cigarette smoke extract (CSE) in vitro and quantified RAGE expression (Fig. 1L–N) and subcellular localization (Fig. 1O, P), respectively. The results confirmed that stimulation with CSE indeed resulted in the upregulation of RAGE in 16-HBE cells. These results collectively confirmed that RAGE expression is increased upon exposure to CS, thereby providing a basis for exploration of RAGE function in the pathogenesis of COPD and its upstream regulatory mechanisms.

### RAGE is essential for CS-induced COPD progression

**Fig. 2:**
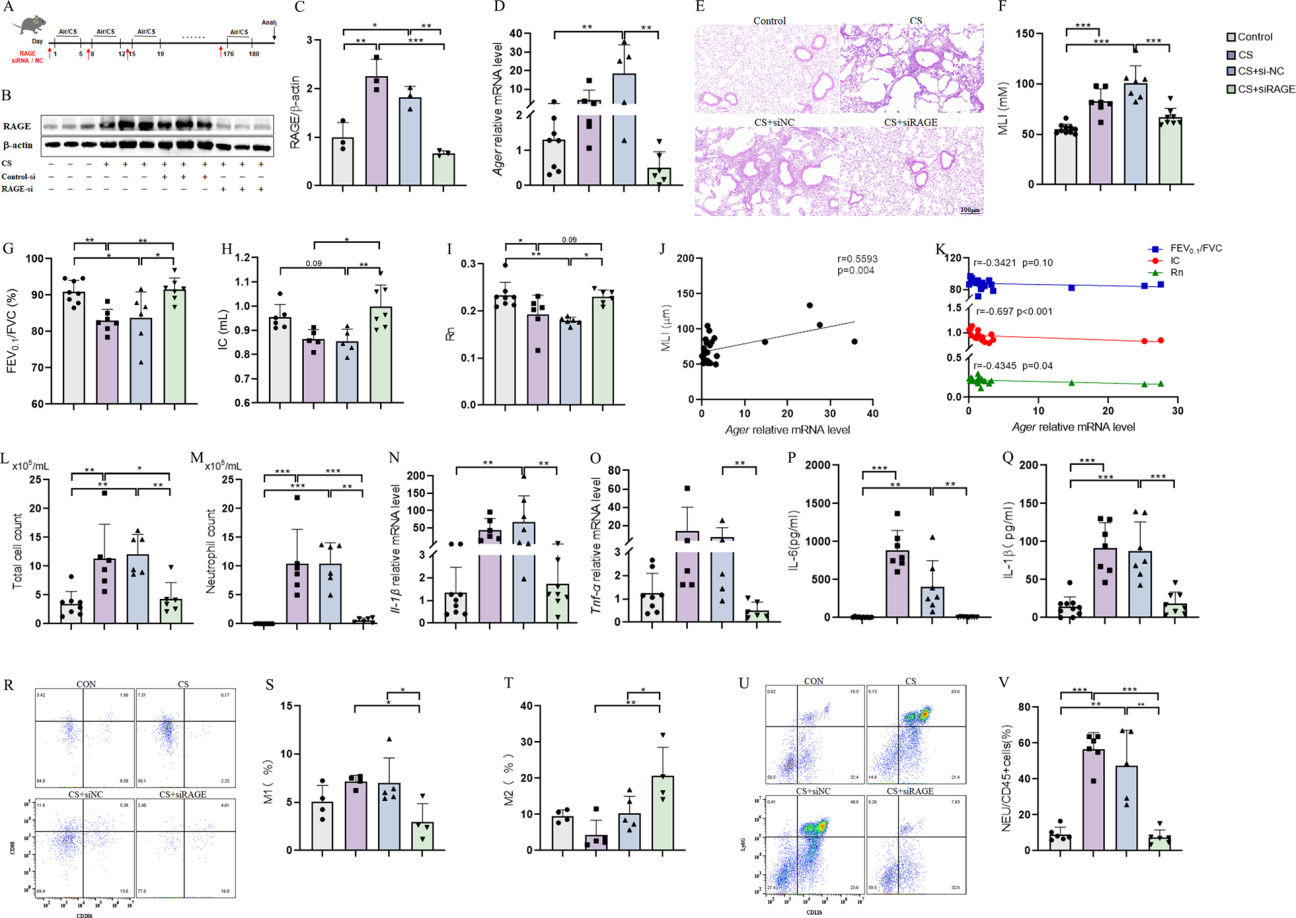
RAGE is essential for CS-induced COPD progression. **A** Flow chart of siRNA administration in chronic obstructive pulmonary disease mice for modeling in vivo; **B**, **C** WB detection of RAGE protein levels in the lung tissue of each group of mice (n = 3); **D** rt-PCR was used to detect the mRNA level of *Ager* in the lung tissue of mice in each group (n = 6–8); **E** Representative images of H&E staining in mouse lung tissue; **F** MLI of mouse lung tissue (n = 6–8); **G**–**I** Mouse lung function indicators FEV_0.1_/FVC, IC and Rn (n = 6–8); **J** Pearson correlation analysis between the MLI of mouse lung tissue and the level of *Ager* mRNA (n = 25); **K** Pearson correlation analysis of FEV_0.1_/FVC, IC, and Rn with *Ager* mRNA levels in mice (n = 25); **L**, **M** statistics of the total number of inflammatory cells and neutrophils in mouse alveolar lavage fluid (BALF) (n = 6–8); **N**, **O** rt-PCR was used to detect the mRNA levels of *IL-1β* and *TNF-α* in the lung tissues of mice in each group (n = 6–8); **P**, **Q** ELISA detection of IL-6 and IL-8 protein levels in mouse lung tissue (n = 6–8); **R**–**T** flow cytometry was used to detect the polarization of macrophage M1 and M2 in mouse lung tissue; **U**, **V** flow cytometry detection of neutrophil infiltration in mouse lung tissue (n = 4–6). The experimental results were independently repeated three or more times, and Data are least squares means ± standard errors. *P < 0.05, **P < 0.01, ***p < 0.001

Since RAGE was significantly increased in response to CS exposure, we continued to explore the role of RAGE in CS-induced COPD progression. We constructed a siRNA by chemical modification with 2-OMe (methoxy), which can persist in vivo for 6 days, to induce RAGE knockdown (KD) , with scrambled siRNA (si-NC) as a control(see experimental scheme in Fig. 2A). The up-regulation of RAGE after CS exposure was clearly attenuated at both mRNA and protein levels in the siRAGE-treated group, whereas it remained significantly elevated in the si-NC group.

H&E staining showed a significant decrease in MLI after siRAGE treatment in COPD mice, while still increasing after si-NC treatment (Fig. 2E, F). Moreover, SCIREQ assays of lung function after CS exposure indicated that RAGE KD resulted in rescue of FEV_0.1_/FVC (Fig. 2G), IC (Fig. 2H) and Rn (Fig. 2I) parameters to levels comparable with untreated controls, significantly higher than that in si-NC-treated CS group. Correlation analysis showed that *Ager* mRNA levels shared a significant positive correlation (r = 0.5593, p = 0.004) with MLI (Fig. 2J), but were significantly negatively correlated with IC (r = 0.697, p < 0.001) and Rn (r = 0.4345, p = 0.04) (Fig. 2K), while there seems to be a negative correlation with FEV_0.1_/FVC (r = 0.3421, p = 0.1). To assess the severity of airway inflammation, we collected BALF from mice in each treatment group to quantify and characterize inflammatory cell populations. We found that both total cell numbers (Fig. 2L) and neutrophil populations (Fig. 2M) were significantly increased after CS exposure compared to that in untreated control mice, whereas total cells and neutrophils detected in BALF of siRAGE COPD mice were similar to that in untreated controls. We also counted the number of macrophages, eosinophils, and lymphocytes, which did not change significantly between groups (Additional file 1: Fig. S1 F–H). In addition, rt-qPCR analysis of *IL-1β* (Fig. 2N) and *TNF-α* (Fig. 2O) mRNA levels in mouse lung and ELISA detection of IL-6 (Fig. 2P) and IL-1β (Fig. 2Q) protein in BALF together showed that inflammatory cytokines were elevated after CS exposure, which could be significantly abolished by siRAGE administration, but not si-NC. In vitro cultured 16-HBE cells, we also observed that siRAGE significantly inhibited the secretion of IL-6 and IL-8 in the supernatant of the cell medium (Additional file 1: Fig. S1I, J), while simultaneously suppressing RAGE (Additional file 1: Fig. S1 C–E). In addition, RAGE specific inhibitor fps-zm1 also plays a significant inhibitory role in the production and secretion of inflammatory factors (Additional file 1: Fig. S1K–P). Flow cytometry-based detection of macrophage polarization and neutrophil infiltration in mouse lung tissue further confirmed that both macrophage M1 polarization (Fig. 2R–T, Additional file 1: Fig. S1Q) and subsequent neutrophil infiltration (Fig. 2U, V, Additional file 1: Fig. S1R) were significantly inhibited in COPD mice with RAGE KD following treatment with CS. These cumulative results indicated that RAGE was essential for the CS-induced airway inflammation in mice with COPD.

### MiR-23a-5p could inhibit the expression of RAGE and was reduced in mice with COPD 

**Fig. 3:**
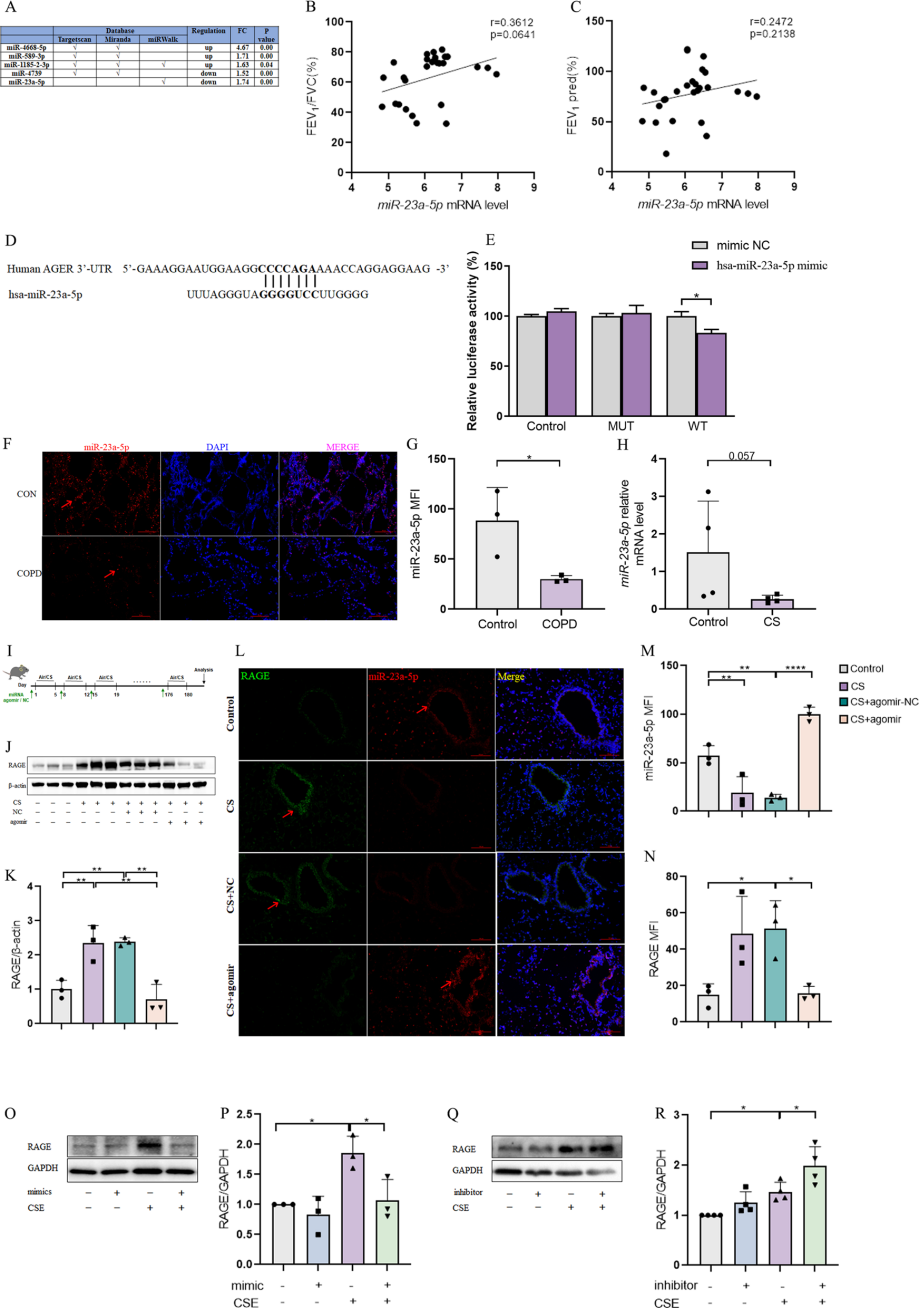
Down-regulated miR-23a-5p can target and decrease RAGE in COPD mice. **A** Differentially expressed microRNAs targeting RAGE were screened through bulk RNAseq analysis and website prediction; **B** Pearson correlation analysis between *miR-23a-5p* mRNA level and FEV_1_/FVC of subjects (n = 28); **C** Pearson correlation analysis between *miR-23a-5p* mRNA level and FEV_1_ pred (%) of subjects (n = 28); **D** schematic diagram of binding sites between *miR-23a-5p* and *AGER*; **E** double luciferase report experiment to verify the binding of miR-23a-5p with AGER (n = 3); **F**, **G** fluorescence in situ hybridization (FISH) assay to detect the expression of miR-23a-5p in mouse lung tissue and analyze the average fluorescence intensity (MFI) (n = 3); **H** rt-PCR was used to detect mRNA levels of *mir-23a-5p* in the lung tissue of control and COPD mice (n = 4); **I** Model construction flowchart; **J**, **K** Western Blotting detection of RAGE protein levels in the lung tissue of mice in each group (n = 3); **L**–**N** fluorescence in situ hybridization (FISH) was used to detect the expression of miR-23a-5p and RAGE in mouse lung tissue and analyze the average fluorescence intensity (MFI) (n = 3); **O**, **P** Western Blotting detection of RAGE protein levels in 16-HBE cells transfected with miR-23a-5p mimics; Q-R: detection of RAGE protein levels in 16-HBE cells transfected with miR-23a-5p inhibitor. The experimental results were independently repeated three or more times, and Data are least squares means ± standard errors. *P < 0.05, **P < 0.01, ***p < 0.001

As a typical post-transcriptional regulatory factor, miRNA was complementary to 3′-UTR of the target gene to inhibit the expression of the target gene. In order to screen for potentially downregulated miRNAs that might target RAGE, we collected peripheral blood from 13 COPD patients and 19 healthy control volunteers (see Table 2) for bulk RNAseq analysis (Fig. 3A). This analysis identified miR-23a-5p as a likely candidate miRNA targeting RAGE that was significantly less abundant in COPD patients than healthy volunteers. Subsequent correlation analysis showed that *miR-23a-5p* transcription was almost positively correlated with FEV_1_/FVC ratio (r = 0.3612, p = 0.0641) (Fig. 3B), as well as FEV_1_ pred (%) (r = 0.2472, p = 0.2138) (Fig. 3C), which suggested that miR-23a-5p may play a role in the pathogenesis of COPD. Following bioinformatic prediction of the nucleotides likely involved in interaction between *miR-23a-5p* and *AGER* (Fig. 3D), we performed dual luciferase reporter assays and found that co-transfection with the vectors harboring the wild-type *AGER* 3′-UTR and an miR-23a-5p mimic resulted in decreased luciferase activity, while no changes in vectors containing a disrupted binding site 3′-UTR variant or the reporter-only control and the miR-23a-5p mimic (Fig. 3E). These results suggested that miR-23a-5p could directly bind to the 3′-UTR of *AGER*. In addition, fluorescent in situ hybridization (FISH) (Fig. 3F–G) and rt-qPCR (Fig. 3H) in lung tissue of COPD model mice showed that miR-23a-5p levels were indeed significantly lower in lungs of COPD mice compared to controls.

**Table 2:** Subjects characteristics

Variables	Total	COPD	Health control	P value
Number	32	13	19	
Age, year	63.91 ± 6.26	65.62 ± 4.07	62.74 ± 7.16	0.214
Sex, M:F(10%M)	17:15	8:5 (61.54%)	9:10 (47.37%)	0.43
Height (cm)	166.1 ± 8.52	166.38 ± 9.57	165.89 ± 8.22	0.878
Weight (Kg)	70.92 ± 12.36	70.92 ± 11.22	70.92 ± 13.78	0.999
Body Mass Index	25.64 ± 3.67	25.58 ± 3.39	25.68 ± 4.06	0.939
FEV_1_/FVC (%)	63.32 ± 15.72	47.98 ± 12.96	74.39 ± 4.74	< 0.0001
FEV_1_ pred (%)	77.29 ± 23.31	58.28 ± 19.26	91.01 ± 15.93	< 0.0001

All continuous data are given as median (IQR) or mean (SD), all categorical data as proportion (%)

Based on these findings, we constructed a miR-23a-5p mimic (agomir) which was administered weekly through tracheal instillation during the COPD model establishment (Fig. 3I). RAGE protein levels were significantly lower in lung tissue after miR-23a-5p agomir treatment, compared to that in CS-only and CS-agomir negative control (NC)mice (Fig. 3J, K). The above FISH assays had a similar trend with Western Blotting (Fig. 3L–N). Investigation of miR-23a-5p regulatory function using miR-23a-5p mimic or the miR-23a-5p inhibitor, in 16-HBE cells in vitro showed that RAGE expression was significantly inhibited in the presence of miR-23a-5p mimics (Fig. 3O, P, Additional file 2: Fig. S2A), but increased upon CSE stimulation in cells treated with the inhibitor (Fig. 3Q, R). Together, these results illustrated that miR-23a-5p, which is downregulated during the pathogenesis of COPD, has a negative regulatory effect on RAGE in vitro and in vivo.

### MiR-23a-5p reduces emphysema and airway inflammation, improving lung function in COPD mice

**Fig. 4:**
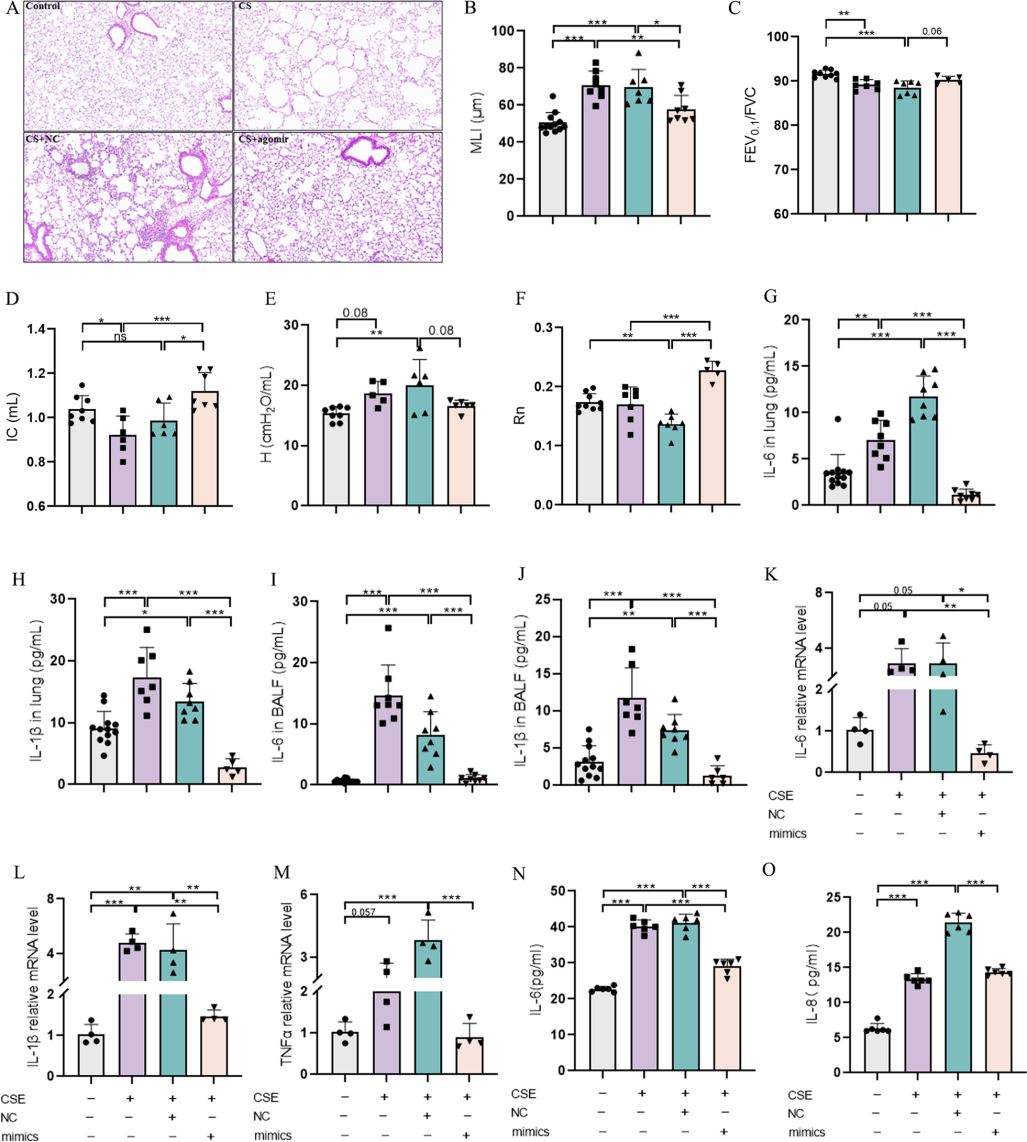
MiR-23a-5p reduced emphysema and airway inflammation in COPD mice, leading to improved lung function. **A** Representative images of the H&E stain in the lung tissue of each group of mice; **B** MLI of mouse lung tissue (n = 6–8); **C**–**F** The levels of FEV_0.1_/FVC, IC, tissue elasticity (**H**) and Rn in different groups (n = 6–8); **G**, **H** ELISA detection of IL-6 and IL-8 protein levels in the lung tissue of mice in different groups (n = 6–8); **I**, **J** ELISA detection of IL-6 and IL-8 protein levels in BALF of different mice (n = 6–8); **K**–**M** rt-PCR was used to detect the mRNA levels of *IL-1β* and *TNF-α* in HBE cells transfected with mimics; **N**, **O** ELISA was used to detect the secretion levels of IL-6 and IL-8 in 16-HBE cells transfected with mimics. The experimental results were independently repeated three or more times, and Data are least squares means ± standard errors. *P < 0.05, **P < 0.01, ***p < 0.001

Given our findings that miR-23a-5p is downregulated in COPD patients and that this miRNA has inhibitory effects on RAGE levels in vivo and in vitro, we next investigated whether miR-23a-5p could potentially confer therapeutic effects in mice with COPD. Evaluation of airway inflammation, alveolar structure, and lung function revealed that treatment with miR-23a-5p agomir was associated with alleviation of CS-induced alveolar structural damage, as indicated by lower MLI values in the CS + miR-23a-5p agomir group compared to the CS-treated control groups (Fig. 4A, B). At the same time, CS-induced abnormalities in indicators of lung function, including FEV_0.1_/FVC (Fig. 4C), Rn (Fig. 4D), IC (Fig. 4E), and H (Fig. 4F) were restored in COPD mice treated with miR-23a-5p agomir. Similarly, ELISA detection of IL-6 and IL-1β protein level in mouse lung tissue and BALF showed that the IL-6 (Fig. 4G, I) and IL-1β (Fig. 4H, J) were significantly inhibited in both lung and BALF of the miR-23a-5p agomir group, indicating that miR-23a-5p exerted an overall anti-inflammatory influence. In addition, we detected the protein level of monocyte chemokine CCL-2 in BALF and lung tissue by ELISA, and the results also found that CCL-2 was significantly increased after CS exposure, while miR-23a-5p agomir could effectively inhibit the protein level of CCL-2 (Additional file 2: Fig. S2B, C), which might inhibit inflammatory cell recruitment.

Further, the miR-23a-5p mimic could effectively suppress the CSE-induced increase in *IL-6* (Fig. 4K), *IL-1β* (Fig. 4L) and *TNF-α* (Fig. 4M) mRNAs in 16-HBE cells. This trend in rt-qPCR data was recapitulated in assays quantifying secreted IL-6 (Fig. 4N) and IL-8 (Fig. 4O) in 16-HBE cells in vitro. These results suggested that negative regulation of RAGE by miR-23a-5p might potentially result in therapeutic effects in COPD.

### CS exposure activates reactive oxygen species (ROS) signaling downstream of miR-23a-5p/RAGE

**Fig. 5:**
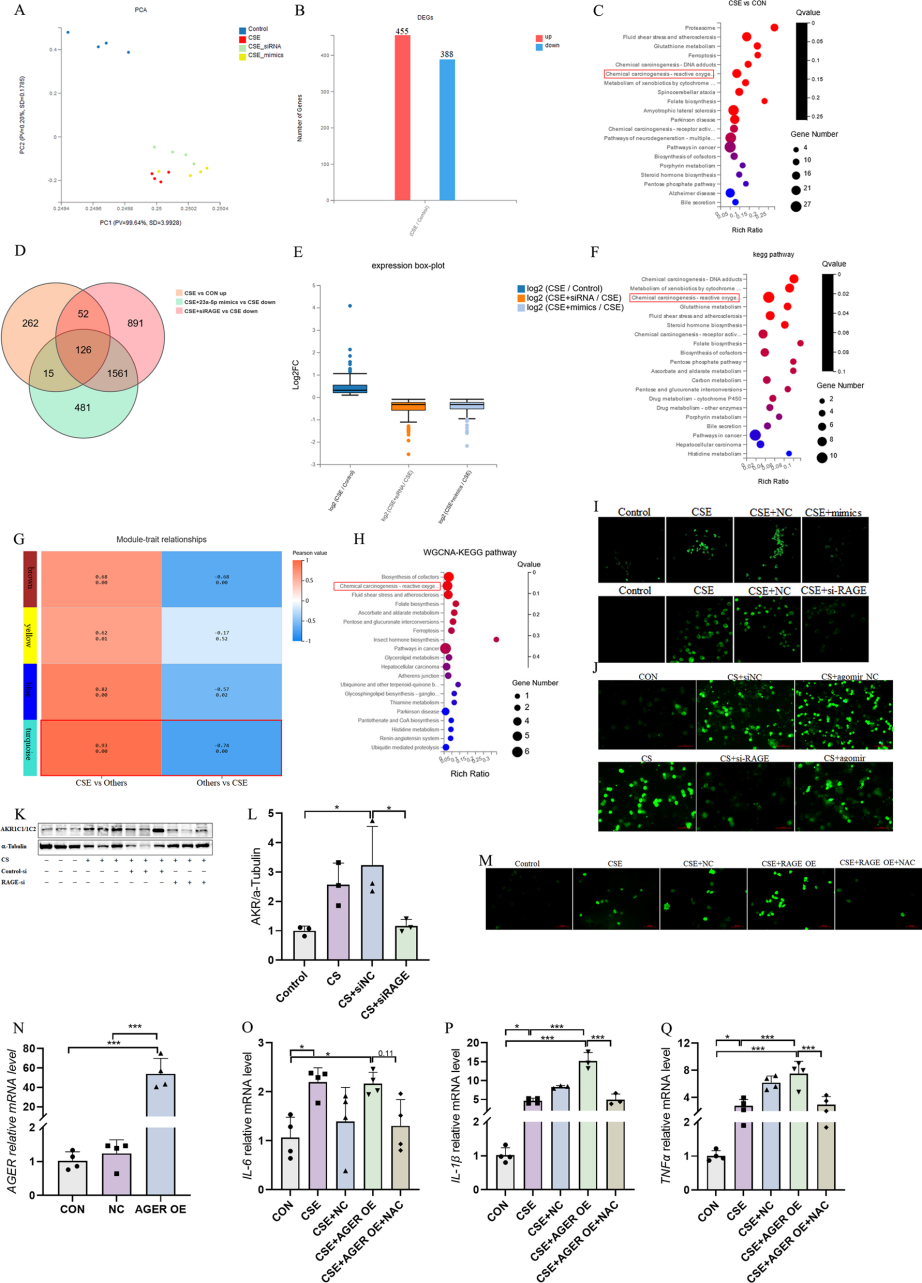
CS exposure activates reactive oxygen species (ROS) signaling downstream of miR-23a-5p/RAGE. **A** Principal component analysis for the expression of genes in different populations of 16-HBE cells; **B** the number of the differentially expressed genes (DEGs) in 16-HBE cells after different treatment; **C** KEGG pathway enrichment analysis of DEGs in the CSE group compared to controls; **D** Venn diagram of DEGs in different groups; **E** boxplots of the distribution of different groups of differential genes in HBE cells; **F** KEGG pathway enrichment analysis of downregulation-DEGs related with siRAGE and miR-23a-5p mimics treatment; G: WGCNA analysis was used to analyze the correlation between different treatments and DEGs in 16-HBE cells. **H** KEGG pathway enrichment analysis of strongly correlated genes in WGCNA analysis; **I** Immunofluorescence was used to detect the production of reactive oxygen species (ROS) after transfection of siRAGE and miR-23a-5p mimics in 16-HBE cells. **J** ROS positive staining was detected by immunofluorescence in alveolar lavage fluid cells from different groups of mice; **K**, **L** WB was used to detect levels of AKR protein in lung tissue of mice. **M** Immunofluorescence was used to detect ROS production in 16-HBE cells transfected with RAGE over-expression plasmids and ROS scavengers. **N** *AGER* mRNA levels were detected by rt-PCR after 16-HBE cells were transfected with a RAGE over-expression (OE) plasmids. **O**–**Q** mRNA levels of *IL-6, IL-1β* and *TNF-α* were detected by rt-PCR after 16-HBE cells transfected with RAGE OE plasmids. The experimental results were independently repeated three or more times, and Data are least squares means ± standard errors. *P < 0.05, **P < 0.01, ***p < 0.001

To further investigate the specific mechanism through which miR-23a-5p/RAGE contributes to the pathogenesis of COPD, we transfected 16-HBE cells with miR-23a-5p mimics or RAGE siRNA, then stimulated the cells with CSE in vitro to collect total RNA for bulk RNAseq analysis (Fig. 5A). Analysis of differentially expressed genes (DEGs) between treatment and control groups identified 455 up-regulated and 388 down-regulated genes in response to CSE treatment (Fig. 5B). Subsequent KEGG pathway enrichment analysis implied that ferroptosis and ROS pathways were significantly enriched among up-regulated genes (Fig. 5C), in which 126 of the upregulated DEGs after CSE treatment were downregulated by miR-23a-5p mimic and RAGE siRNA (Fig. 5D, E).

KEGG pathway enrichment analysis of these 126 genes showed that ROS signaling was still significantly enriched (Fig. 5F). WGCNA analysis identified a significant positive correlation between ROS pathway-related genes and CSE exposure, but that this correlation was inhibited by miR-23a-5p mimics or RAGE siRNA (Fig. 5G, H), suggesting that ROS signaling downstream of miR-23a-5p/RAGE may contribute to COPD pathogenesis. To verify these results, we assessed ROS level in cells isolated from BALF of COPD and control mice in vivo. The results showed that the proportion of ROS-positive cells was markedly higher in the BALF of CS-only control mice than in the RAGE siRNA control group (Fig. 5J). Immunostaining for ROS in 16-HBE cells in vitro showed that ROS was decreased in cells treated with CSE plus miR-23a-5p mimics or RAGE siRNA compared with CSE group (Fig. 5I). Similarly, administration of miR-23a-5p agomir or RAGE siRNA in COPD mice in vivo resulted in significantly reducing the fraction of ROS-positive cells in BALF compared with CS-stimulated controls (Fig. 5J).

Based on our above RNAseq results, we next used Western blotting to detect a key molecule in ROS signaling, aldosterone reductase (AKR), which revealed that AKR1C1/1C2 protein levels increased significantly after smoke exposure, while expression of RAGE siRNA could suppress AKR1C1/1C2 protein levels (Fig. 5K, L). To further confirm that ROS signaling downstream of miR-23a-5p/RAGE played a role in COPD-associated inflammation, we constructed a RAGE overexpression plasmid (OE), which was subsequently transfected, or a vector control, into 16-HBE cells (Fig. 5N). Staining for ROS indicated that RAGE overexpression resulted in significantly increased ROS signal, which could be reversed by treatment with the ROS scavenger, acetylcysteine (NAC) (Fig. 5M). In addition, we also quantified mRNA levels of the inflammatory cytokines, *IL-6* (Fig. 5O)*, IL-1β* (Fig. 5P), and *TNF-α* (Fig. 5Q), and found that expression of these cytokines was also increased along with ROS levels in cells overexpressing RAGE, thus supporting the pro-inflammatory effects of RAGE, which could be at least partially inhibited by NAC treatment. Based on the above results, it was reasonable to hypothesize that the ROS signaling pathway might potentially contribute to COPD pathogenesis after CS exposure downstream of miR-23a-5p/RAGE.

### The MAPK signaling pathway may be involved in the miR-23a-5p/RAGE regulatory module in the pathogenesis of COPD

**Fig. 6:**
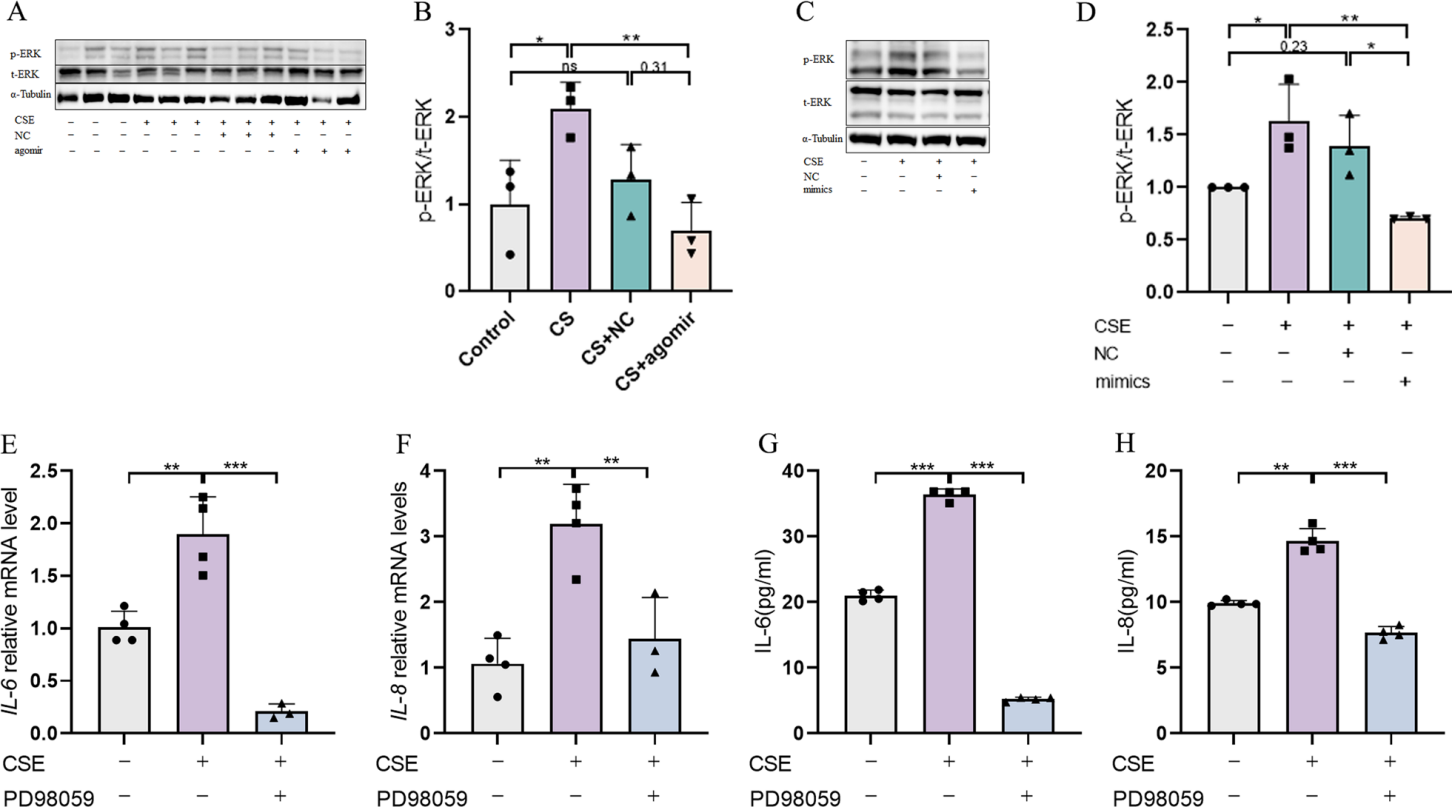
The MAPK signaling pathway may be involved in the miR-23a-5p/RAGE regulatory module in the pathogenesis of COPD. **A**, **B** Western Blotting detection of ERK phosphorylation levels in lung tissues of different groups of mice (n = 3); **C**, **D** Western Blotting detection of ERK phosphorylation levels in HBE cells transfected with miR-23a-5p mimics; **E**, **F** rt-PCR detection of mRNA levels of IL-6 and IL-8 in HBE cells after ERK inhibitor administration; G-H: ELISA detection of IL-6 and IL-8 secretion levels in HBE cells after ERK inhibitor (PD98059) administration. The experimental results were independently repeated three or more times, and Data are least squares means ± standard errors. *P < 0.05, **P < 0.01, ***p < 0.001

As numerous studies have reported that ERK signaling is activated and initiates inflammatory signaling in response to ROS [26, 27], we used Western blotting assays to evaluate ERK phosphorylation levels in lung tissue of mice exposed to CS. The results showed that phosphorylation of ERK was significantly increased after smoke exposure, and that this increase was significantly inhibited in mice expressing miR-23a-5p agomir (Fig. 6A, B). A similar phenomenon was observed in CSE-stimulated 16-HBE cells in vitro*,* which displayed significantly greater ERK phosphorylation levels than untreated controls, whereas this CSE-induced increase in p-ERK was abolished in cells transfected with miR-23a-5p mimics (Fig. 6C, D). In addition, treatment with the ERK-specific blocker, PD98059, prior to CSE treatment in 16-HBE cells resulted in effectively blocking CSE-induced production and secretion of IL-6 (Fig. 6E–G) and IL-8 (Fig. 6F–H). At the same time, we also saw that siRAGE inhibited the phosphorylation of p38 MAPK after CSE exposure (Additional file 3: Fig. S3A, B), and the use of JNK inhibitor (SP600125) and p38 inhibitor (SB203580) could inhibit the secretion of inflammatory factors to varying degrees (Additional file 3: Fig. S3C, D). The above results suggested that miR-23a-5p/RAGE and ROS signaling likely regulate the pathogenesis of COPD via activation of MAPK.

## Discussion

In this study, we constructed mouse and epithelial cell models of COPD through cigarette smoke exposure and evaluated the model through lung function tests, H&E staining, MLI calculations, and inflammatory factor and inflammatory cell measurement. We found that RAGE expression significantly increases following exposure to CS in COPD patients and mice, and that this upregulation can play a key role in the progression of COPD. This finding provides strong evidence for us to pursue the following studies on the mechanisms underlying the role of RAGE in the pathogenesis of COPD, and to search for its upstream miRNA. RAGE is a multi-ligand pattern-recognition receptor, and many of the ligands of RAGE contribute to the pathogenesis of COPD by binding to RAGE [10, 11, 14]. However, few studies have focused on the role of miRNA in the pathogenesis of COPD by targeting RAGE. We confirmed that miR-23a-5p functions as an upstream regulator of RAGE through sequencing and in vitro and in vivo models of COPD, which collectively illustrated that miR-23a-5p may potentially confer therapeutic effects to alleviate COPD. Finally, our study confirms that the miR-23a-5p-RAGE regulatory module plays a role in COPD via downstream ROS signaling and the MAPK pathway, suggesting a pathway mechanism for activation or suppression of inflammatory response to cigarette smoke in lung tissue.

COPD is a heterogeneous lung disease characterized by chronic respiratory symptoms (e.g., dyspnea, cough, sputum production) caused by abnormalities in the airway (e.g., bronchitis or bronchiolitis), and/or alveoli (e.g., emphysema), leading to persistent and progressive obstruction of airflow [28]. COPD is a major cause of morbidity and mortality worldwide and imposes an increasingly severe social and economic on affected individuals, their families, and public healthcare systems [29]. The *AGER* gene has long been known to play a pathological role in a variety of airway diseases, including COPD, and a number of recent studies have further clarified the role of RAGE and its ligands in the pathogenesis of COPD [18, 30]. Our current research revealed that RAGE was increased in COPD patient and mouse, while inhibition of RAGE can reduce the severity of COPD, include alveolar destruction, inflammation, and lung function, all of this shed a light on the importance of RAGE in the pathogenesis of COPD. Therefore, the search for a miRNA that can specifically bind to and inhibit RAGE has become particularly important.

Other recent reports have shown that miRNAs play an essential role in the occurrence and development of inflammatory airway diseases [31, 32]. For instance, several studies have confirmed that environmental pollutants, including PM_2.5_, may alter the expression profile of miRNAs, resulting in pathogenic effects in humans [33, 34]. In this study, we characterized the miRNA and mRNA transcriptomic profiles of mononuclear cells in the peripheral blood of COPD patients and observed the aberrant expression of multiple miRNA molecules in COPD. MiR-23a-5p was selected for further study based on its predicted sequence motif for binding RAGE. Thus, our study represents, to our knowledge, the first report describing miR-23a-5p function as a regulator of RAGE in the pathogenesis of COPD. Treatment with miR-23a-5p agomir in vivo significantly improved lung function in mice with COPD, an important indicator of COPD diagnosis and severity. FEV_0.1_/FVC, IC and other indicators decreased after CS exposure, which could be recovered with miR-23a-5p agomir. COPD is a small airway disease, so Rn, as an indicator of atmospheric airway resistance, does not show a clear trend in the pathogenesis of COPD. In addition, miR-23a-5p agomir also significantly reduced lung tissue destruction and lung inflammation due to CS exposure, as reflected by MLI and inflammatory factor detection, suggesting new and potentially effective therapeutic strategies for improving the generally poor outcomes of COPD treatment with current options.

Although our study provides several advances in understanding the regulation of COPD progression, some limitations of our study should be kept in mind. First, as COPD is defined as a small airway disease, we mainly focus on the role of RAGE in airway epithelial cells. Due to technical limitations, we were unable to obtain alveolar epithelial cells for in vitro investigations of the miR-23a-5p/RAGE regulatory module. Overcoming this technical obstacle will facilitate further detailed experiments in future studies. Second, our screening of mirnas is dominated by those derived from peripheral blood rather than lung tissue. Although we subsequently verified the expression of miR-23a-5p and its regulatory effect on RAGE in mouse lung tissue, this may make our results less convincing. Third, our exploration of the downstream targets/effects of miR-23a-5p/RAGE do not likely encompass the full suite of RAGE-responsive mechanisms involved in COPD development, which will also require further systematic investigation in future work. In addition, only an agomir of miR-23a-5p was used for in vivo experiments, with no verification of miR-23a-5p therapeutic effects on COPD by chemical inhibitors or other means. Further experimental evidence defining these effects will be obtained in the future.

In summary, our study details an upstream regulator of RAGE, and further defines its downstream inflammatory signaling pathways in COPD, which can guide the development of effective drugs that target RAGE to treat COPD.

## Conclusion

Our study confirmed that miR-23a-5p, as the upstream regulatory negative factor of RAGE, inhibits the activation of the downstream ROS and MAPK signaling pathway, significantly improves the pathological changes and lung function induced by cigarette smoke exposure, and may become an effective drug for the treatment of COPD targeting RAGE.

### Supplementary Information

**Additional file 1: Figure S1.** Inhibition of RAGE suppressed cellular inflammation. A: Representative images of haematoxylin and eosin (H&E) staining in mouse lung sections from different groups; B: Mean linear interval (MLI) of mouse lung tissues (n = 6–8); C: rt-qPCR was used to detect the mRNA level of AGER in 16-HBE cells transfected with siRAGE; D, E: Western Blotting was used to detect the protein level of RAGE in 16-HBE cells transfected with siRAGE; F–H: Statistics of the number of macrophages, eosinophils and lymphocytes in mouse alveolar lavage fluid (BALF) (n = 6–8); I, J: ELISA was used to detect the levels of IL-6 and IL-8 in the culture medium of 16-HBE cells transfected with siRAGE; K–O: rt-qPCR was used to detect the mRNA levels of *AGER*, *IL-6*, *IL-8*, *IL-1β*, and *TNFα* in cells treated with the RAGE-specific inhibitor, named fps-zm1; P: ELISA was used to detect the secretion of IL-6 in the culture medium supernatant after fps-zm1 treatment; Q, R: The gating strategy of flow cytometry for macrophages and neutrophils. The experimental results were independently repeated three or more times, and Data are least squares means ± standard errors. *P < 0.05 **P < 0.01, ***p < 0.001.

**Additional file 2: Figure S2.** The effect of miR-23a-5p on monocyte chemokine CCL-2. A: rt-qPCR was used to detect the mRNA level of AGER in 16-HBE cells transfected with miR-23a-5p mimics; B, C: The C-Cmotifchemokineligand2 (CCL-2) protein levels in lung and BALF of different mice were measured by ELISA (n = 6–8). The experimental results were independently repeated three or more times, and Data are least squares means ± standard errors. *P < 0.05, **P < 0.01, ***p < 0.001.

**Additional file 3: Figure S3.** The role of MAPK in RAGE-related inflammation. A, B: Western Blotting was used to detect the phosphorylation level of p38 in 16-HBE cells transfected with siRAGE; C, D: Detection of the effect of MAPK signaling pathway inhibitors on the concentration of IL-6 and IL-8 in the culture medium. The experimental results were independently repeated three or more times, and Data are least squares means ± standard errors. *P < 0.05; **P < 0.01; ***p < 0.001.

## Data Availability

All about the data showed in the study are included in the original article or in supplementary materials, further information can be directed to the corresponding authors.
