# Supplementary figures and images for: MiR-23a-5p alleviates chronic obstructive pulmonary disease through targeted regulation of RAGE-ROS pathway
Source: Respir Res. 2024 Feb 20;25:93. doi: 10.1186/s12931-024-02736-y (PMC10880325; doi:10.1186/s12931-024-02736-y)

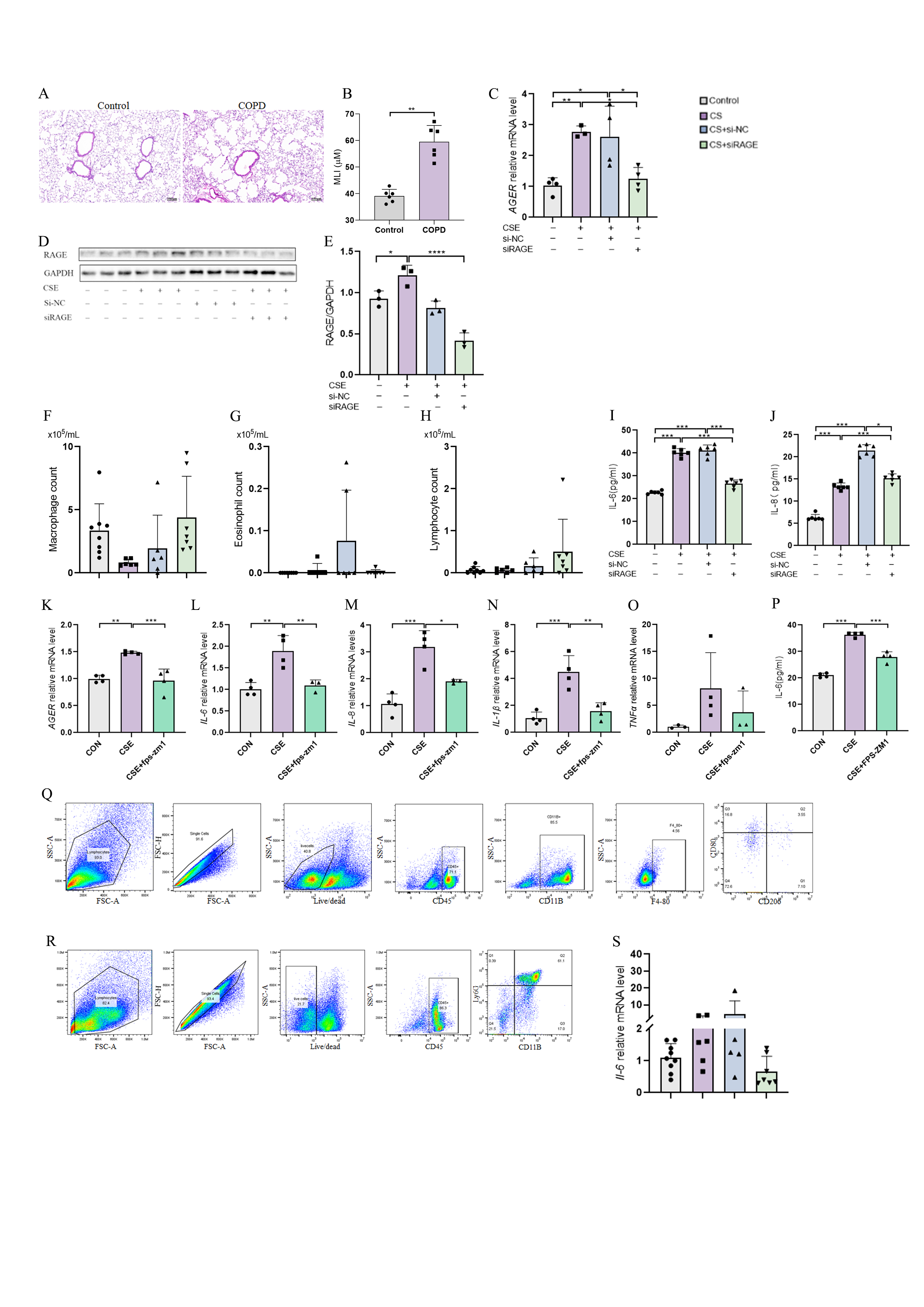

Supplement: Supplementary file 1 — Additional file 1: Figure S1. Inhibition of RAGE suppressed cellular inflammation. A: Representative images of haematoxylin and eosin (H&E) staining in mouse lung sections from different groups; B: Mean linear interval (MLI) of mouse lung tissues (n = 6–8); C: rt-qPCR was used to detect the mRNA level of AGER in 16-HBE cells transfected with siRAGE; D, E: Western Blotting was used to detect the protein level of RAGE in 16-HBE cells transfected with siRAGE; F–H: Statistics of the number of macrophages, eosinophils and lymphocytes in mouse alveolar lavage fluid (BALF) (n = 6–8); I, J: ELISA was used to detect the levels of IL-6 and IL-8 in the culture medium of 16-HBE cells transfected with siRAGE; K–O: rt-qPCR was used to detect the mRNA levels of AGER, IL-6, IL-8, IL-1β, and TNFα in cells treated with the RAGE-specific inhibitor, named fps-zm1; P: ELISA was used to detect the secretion of IL-6 in the culture medium supernatant after fps-zm1 treatment; Q, R: The gating strategy of flow cytometry for macrophages and neutrophils. The experimental results were independently repeated three or more times, and Data are least squares means ± standard errors. *P < 0.05 **P < 0.01, ***p < 0.001. [file 12931_2024_2736_MOESM1_ESM.tif]

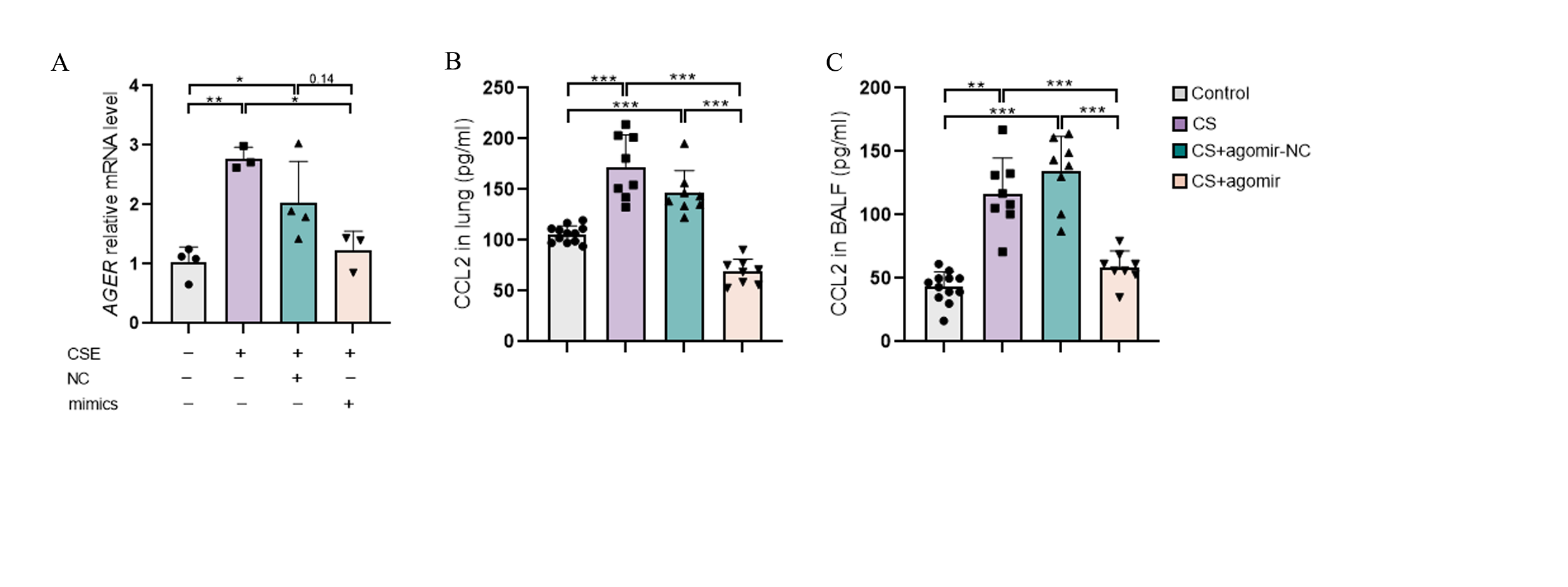

Supplement: Supplementary file 2 — Additional file 2: Figure S2. The effect of miR-23a-5p on monocyte chemokine CCL-2. A: rt-qPCR was used to detect the mRNA level of AGER in 16-HBE cells transfected with miR-23a-5p mimics; B, C: The C-Cmotifchemokineligand2 (CCL-2) protein levels in lung and BALF of different mice were measured by ELISA (n = 6–8). The experimental results were independently repeated three or more times, and Data are least squares means ± standard errors. *P < 0.05, **P < 0.01, ***p < 0.001. [file 12931_2024_2736_MOESM2_ESM.tif]

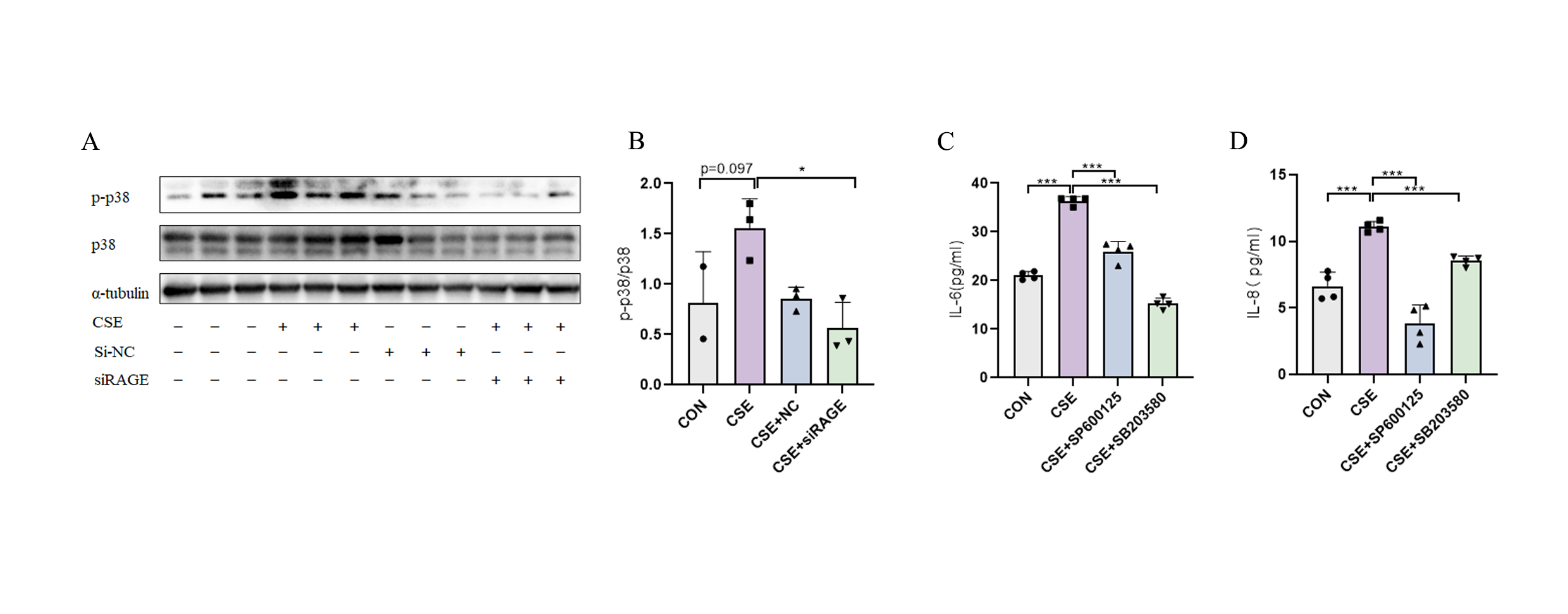

Supplement: Supplementary file 3 — Additional file 3: Figure S3. The role of MAPK in RAGE-related inflammation. A, B: Western Blotting was used to detect the phosphorylation level of p38 in 16-HBE cells transfected with siRAGE; C, D: Detection of the effect of MAPK signaling pathway inhibitors on the concentration of IL-6 and IL-8 in the culture medium. The experimental results were independently repeated three or more times, and Data are least squares means ± standard errors. *P < 0.05; **P < 0.01; ***p < 0.001. [file 12931_2024_2736_MOESM3_ESM.tif]
